# Effects of the Molecular Weight of Hyaluronic Acid in a Carbon Nanotube Drug Delivery Conjugate

**DOI:** 10.3389/fchem.2020.578008

**Published:** 2020-12-14

**Authors:** Silvia Arpicco, Michał Bartkowski, Alessandro Barge, Daniele Zonari, Loredana Serpe, Paola Milla, Franco Dosio, Barbara Stella, Silvia Giordani

**Affiliations:** ^1^Department of Drug Science and Technology, University of Torino, Turin, Italy; ^2^School of Chemical Sciences, Dublin City University (DCU), Dublin, Ireland

**Keywords:** carbon nanotubes, hyaluronic acid, targeted drug delivery, cancer, phospholipids, CD44 receptor, nanocarrier

## Abstract

Hyaluronic acid (HA) is a ubiquitous biopolymer involved in many pathophysiological roles. One HA receptor, the cluster of differentiation CD44 protein, is often overexpressed in tumor cells. As such, HA has attracted considerable interest in the development of drug delivery formulations, given its intrinsic targetability toward CD44 overexpressing cells. The present study is focused on examining the correlation of HA molecular weight with its targetability properties. A library of conjugates obtained by linking the amino group of the phospholipid 1,2-dimyristoyl-*sn*-glycero-3-phosphoethanolamine (DMPE) to the carboxylic residues of HA of different molecular weight (6.4, 17, 51, 200, and 1,500 kDa) were synthesized and fully characterized. The HA-DMPE conjugates were then used to non-covalently functionalize the highly hydrophobic single-walled carbon nanotubes (CNT), and further encapsulate the anticancer drug doxorubicin (DOX). Our results show that the complexes DOX/CNT/HA-DMPE maintain very good and stable dispersibility. Drug release studies indicated a pH-responsive release of the drug from the nanocarrier. Cell viability tests demonstrated that all HA modified CNTs have good biocompatibility, and specific targeting toward cells overexpressing the CD44 receptor. Among all the molecular weights tested, the 200 kDa HA showed the highest increase in cellular uptake and cytotoxic activity. All these promising attributes make CNT/HA_200_-DMPE a “smart” platform for tumor-targeted delivery of anticancer agents.

## Introduction

Hyaluronic acid (HA) is a linear polysaccharide consisting of repeating β-1,4-D-glucuronic acid and β-1,3-*N*-acetyl-D-glucosamine disaccharide units. The disaccharide unit can repeat thousands of times, achieving high molecular weights (MW) of over 5,000 kDa. HA represents the main component of the extracellular matrix and is ubiquitously distributed in vertebrate tissues. In the human body, predominantly in the skin, the total HA is about 15 g, with a rapid daily turnover achieved through the activity of hyaluronidases (Stern, 2004). Regarding the HA physiologic role, it plays both a structural role, for example, in the skin due to unique hydrodynamic properties, as well as cell signaling role, for example, during the dynamic cell processes of morphogenesis and inflammation. A large amount of HA in the extracellular matrix of the central nervous system contributes to brain hydration (Perkins et al., 2017). In the lungs, it contributes to the mechanical properties of tissues, such as elasticity, while in the cardiovascular system, HA is involved in several pathophysiological conditions (Fischer, 2019).

To exert its biological properties, HA requires specific interactions with several cell receptors, such as CD44, RHAMM, HARE, and LYVE-1, to trigger specific signaling pathways inside cells. The more representative cell-binding receptor of HA is the cluster of differentiation protein CD44. The interaction of HA with this protein is involved in physiological and pathological processes, particularly in repair mechanisms. Nevertheless, the CD44 receptor is also associated with human cancer, particularly during tumor invasion and metastasis (Toole, 2009). Different therapeutic treatments exploit HA, such as in regenerative, aesthetic, and orthopedic medicine. Due to its intrinsic properties, HA attracts interest in the development of drug delivery agents (Dosio et al., 2016; Passi and Vigetti, 2019). This natural polymer combines useful aspects suitable for a drug delivery nanocarrier, such as its high-water solubility, non-immunogenicity, biocompatibility, and biodegradability.

Furthermore, the functional groups of HA can be easily modified by chemical reactions as its structure is based on the presence of β linkages, which allow for the bulky groups (the hydroxyls, the carboxylate moiety, and the anomeric carbon on the adjacent sugar) to be in sterically favorable equatorial positions. As such, HA can be easily functionalized with other materials, inferring new properties, and improving the existing aspects of the biopolymer. For instance, fluorescence properties can be introduced to HA by functionalizing the polymer with a fluorophore tag. HA could also be used to functionalize carbon nanoparticles, improving their dispersibility properties (d'Amora et al., 2020). Moreover, by exploiting specific enzymatic reactions, HA with precise different molecular weight and properties can be obtained (Valcarcel et al., 2020).

Among the inorganic nanomaterials, carbonaceous nanomaterials are attracting interest due to their excellent mechanical, thermal, and optical properties. Their inert composition is highly beneficial, making them biocompatible and thus highly desirable for biological applications, although with poor aqueous dispersibility (Saleem et al., 2018). Among the carbon-based materials, the nanotubes (CNTs) (Movia and Giordani, 2012; Chen et al., 2017; Mahajan et al., 2018) have demonstrated biocompatibility and they possess a hollow construction and tunable chemistry that provide interesting opportunities for use as delivery systems. The drugs can be loaded onto the CNT surface by a covalent bond, through a cleavable linker or complexation via π-π interactions and adsorption of charged surfactants (Szleifer and Yerushalmi-Rozen, 2005). Many anticancer drugs have been linked with CNTs, such as epirubicin, doxorubicin, methotrexate, quercetin, paclitaxel, gemcitabine, irinotecan, and porphyrins (Bianco et al., 2008; Wong et al., 2013). In an attempt to reduce the high aggregation tendency of pristine CNTs and to improve water and biological media dispersibility, several coating strategies have been developed. From collagen to proteins, polymers, pegylated lipids, and block copolymers have shown to serve as excellent wrapping materials for the non-covalent functionalization of CNTs as a result of π-π stacking and van der Waals interactions. Among these wrapping strategies, HA has demonstrated very beneficial results in dispersing CNTs loaded with doxorubicin (Datir et al., 2012; Cao et al., 2015) and gemcitabine (Prajapati et al., 2019). Given the aforementioned modifiability of the polymer, HA can be coupled to various surfactants, such as 1,2-dimyristoyl-*sn*-glycero-3-phosphoethanolamine (DMPE), which could further improve its dispersibility properties, allowing for increased dispersion of CNTs (Di Crescenzo et al., 2011a,b).

It has been found that the MW of HA regulates its diverse physio-pathological functions (Misra et al., 2015). A similar relationship has also been observed in nanostructures decorated with HA, where MW affected the targeting efficiency; the impact of HA MW on grafted liposomes have been reported by several authors (Arpicco et al., 2013; Mizrahy et al., 2014; Qhattal et al., 2014; Zhong et al., 2019) where the liposomal cellular uptake was improved with increasing of HA MW (5–8 <10–12 <175–350 kDa). Consequently, improved cytotoxic and *in vivo* activity against cancer models have been observed. Li et al. demonstrated that iron oxide nanoparticles coated with 31 kDa HA showed a better targeting ability than lower MW (Li et al., 2014).

Nevertheless, a systematic evaluation of the role of HA MW in increasing dispersibility and imposing targeting activity on CNTs has not yet been investigated. In the present study, we investigate the relationship of HA MW on dispersibility and specific targeting effect of functionalized CNTs, in order to provide a useful guideline for designing similar and other carbon-based HA-decorated nanostructures.

Based on our previous studies, HA-phospholipids conjugates made with five different MW HA were prepared and characterized. Exploiting the strong interaction of phospholipid acyl chains with the surface of CNTs, stable and dispersible derivatives have been obtained. Then, the cationic anthracycline antibiotic doxorubicin was loaded onto the nanotubes via a π-π stacking interaction. Furthermore, the different nanostructures have been investigated for their physiochemical and pharmaceutical properties, evaluating their cellular active targeting efficacy on CD44^+^ and CD44^−^ cell lines.

## Materials and Methods

### Materials

#### Single-Walled Carbon Nanotubes

Single-walled carbon nanotubes (CNTs), 100–1,000 nm in length and 0.8–1.2 nm in diameter, were obtained from Unidym Inc. (CA, USA).

#### Phospholipids

The phospholipids 1,2-dimyristoyl-*sn*-glycero-3-phosphoethanolamine (DMPE) and 1,2-dipalmitoyl-*sn*-glycero-3-phosphoethanolamine (DPPE) were provided by Avanti Polar Lipids, distributed by Sigma-Aldrich S.r.l. (Milan, Italy).

#### Sodium Hyaluronate

Sodium hyaluronate (HA) of different molecular weights: 6.4 kDa (HA_6.4_), 17 kDa (HA_17_), 51 kDa (HA_51_), 200 kDa (HA_200_), and 1,500 kDa (HA_1500_), was purchased from Lifecore Biomedical (MN, USA).

#### Other

Doxorubicin (DOX) hydrochloride was purchased from APAC Pharmaceutical (MD, USA). Fluoresceinamine, isomer I, 1-ethyl-3-[3-(dimethylamino)-propyl]-carbodiimide (EDAC), Fetal Bovine Serum (FBS), Dulbecco's Modified Eagle Medium (DMEM), Roswell Park Memorial Institute (RPMI) 1640 Medium, and all other chemicals, cell culture media, and supplements were sourced from Sigma-Aldrich S.r.l.

### Synthesis and Functionalization

#### Preparation of HA-DMPE and HA-DPPE

The HA-DMPE and HA-DPPE conjugates were prepared following our previously described method (Surace et al., 2009), with slight modifications. Briefly, a mixture of 20 mg of HA and 10 mg of EDAC was dissolved in 5 mL of distilled water. The pH was adjusted to 4 with 0.1 N HCl, and the solution was stirred for 2 h at 37°C. The activated HA was then added to a lipid film of DMPE or DPPE (the phospholipid molar excess was ×25 for HA_6.4_, HA_17_, and HA_51_; ×50 for HA_200_; and ×70 for HA_1500_). The pH was adjusted to 8.4 with a 0.1 M borate buffer (pH 9.5), and the suspension was sonicated at 37 kHz for 30 min. The mixture was stirred for 24 h at 37°C and then centrifuged at 6,000 rpm for 10 min. The precipitate was eliminated, and the supernatant was collected and purified by dialysis for 48 h at 4°C against distilled water using a Spectra/Por® regenerated cellulose membrane (Spectrum, Breda, The Netherlands) with molecular cut-off (MWCO) of 3,500 Da (for HA_6.4_) and 12,000–14,000 Da (for remaining HA MWs) to remove unreacted phospholipids, EDAC, and other by-products. The conjugation reactions were monitored by thin-layer chromatography (TLC) on F_245_ silica gel pre-coated sheets (Merck, Milan, Italy). The purification was confirmed by TLC using chloroform/methanol (70:30 v/v); after migration of the mobile phase, the sheets were exposed to iodine vapors, a solution of molybdenum blue, and ninhydrin (2,2-dihydroxyindene-1,3-dione) solution (2% in ethanol). The conjugates were lyophilized and analyzed by ^1^H nuclear magnetic resonance (^1^H-NMR). The amount of phospholipid linked to HA in the different conjugates was quantified after resuspension in distilled water by a phosphate assay after disruption with perchloric acid (Bartlett, 1959).

#### Preparation of f-HA and f-HA-DMPE

Labeling of HA with fluoresceinamine (f-HA) was carried out as previously described, with minor modifications (de la Fuente et al., 2008). Briefly, a solution composed of 12.5 mg of fluoresceinamine in 0.25 mL of DMSO, 12.5 μL of acetaldehyde and 12.5 μL of cyclohexyl isocyanide was added to a solution of 25 mg HA in 20 mL of deionized water and 20 mL of DMSO, and the pH was adjusted to 4.5 with 0.1 N HCl. Then, the reaction was stirred at room temperature in darkness for 12 h. The f-HA was precipitated with a saturated solution of NaCl and ice-cold ethanol, collected by centrifugation, suspended in and extensively dialyzed against deionized water, lyophilized, and stored at −20°C in the dark before use. The f-HA was used to prepare f-HA-DMPE following the same procedure described for the preparation of HA-DMPE. Fluoresceinamine labeled HA-DMPE conjugates were used to functionalize CNT for the further cellular uptake experiments.

#### Preparation of DOX/CNT and DOX/CNT/HA-DMPE

DOX was non-covalently loaded onto the CNT and CNT/HA-DMPE to make DOX/CNT and DOX/CNT/HA-DMPE, respectively. In this regard, 1 mg of CNTs were suspended in 2 mL of the different MW HA-DMPE conjugates solutions (1 mg/mL in PBS pH 7.4) and bath sonicated for 3 h. Then, a range of 0.5–1.5 mL of DOX solutions (1 mg/mL in PBS pH 7.4) was added in order to determine the appropriate amount of drug that can be loaded onto the CNT. The mixture was then bath sonicated for 2 h at 37°C and the DOX/CNT/HA-DMPE were purified by repeated filtering and washing with PBS (Centrisart® I, MWCO 100 kDa, Sigma-Aldrich) to remove the unbound products. To remove the excess HA-phospholipids obtained starting from HA_200_ and HA_1500_, the suspension was centrifuged at 11,000 rpm for 30 min, and the pellet was recovered. The amount of unloaded DOX was quantified by measuring the absorbance of the supernatants at 480 nm (Beckman 730 spectrophotometer Beckman Coulter, Milan, Italy). The percentage of HA associated with the CNTs was determined by the carbazole assay (Bitter and Muir, 1962).

### Chemico-Physical Characterization

#### Nuclear Magnetic Resonance

^1^H nuclear magnetic resonance (NMR) spectra were recorded on a Bruker Avance 300 spectrometer (Karlsruhe, Germany) operating at 7T. Samples were dissolved in D_2_O.

#### Transmission Electron Microscopy

Transmission electron microscopy (TEM) images were recorded with a JEM 3010 ultrahigh-resolution analytical electron microscope (JOEL) at an accelerating voltage of 300 kV. TEM grids were prepared by spreading a droplet of the sample solution in deionized water on a copper grid coated with a lacey carbon film.

#### Thermogravimetric Analysis

Thermal gravimetric analysis (TGA) was carried out on a TGA 4000 System (PerkinElmer, Waltham, MA, USA). The sample was heated in argon from 50 to 800°C at a rate of 10°C/min.

#### Zeta Potential

The zeta potential (ZP) of all materials was investigated using a Nanosizer Nano Z (Malvern, UK). Measurements were taken in triplicate at 25°C, and the Smoluchowski equation was applied.

#### UV-Vis Absorption Spectroscopy

Absorption spectroscopy analyses were performed on a Shimadzu UV-1800 spectrophotometer using 1 cm path-length quartz cuvettes. Solutions at 1 mg/mL of all the analyzed samples (DOX, HA-DMPE and DOX/CNT/HA-DMPE) were diluted in deionized water to achieve a final concentration of 100 μg/mL. After sonication for 15 min at 37 kHz, solutions at 10, 20, and 50 μg/mL were prepared for the analyses.

#### Fluorescence Spectroscopy

Fluorescence spectroscopy analyses were conducted on an LS-55 spectrofluorometer (PerkinElmer). Sample solutions were prepared at 1 mg/mL and diluted in deionized water to achieve a final concentration of 100 μg/mL. After sonicating the sample for 15 min at 37 kHz, 10 and 20 μg/mL solutions were prepared for the analysis.

#### Stability Studies

The stability of DOX/CNT and DOX/CNT/HA-DMPE at different HA MW was assessed in different biological media. The media used to perform this experiment were commercially available, and included: deionized water, PBS, RPMI 1640, 1640 + 10% FBS, DMEM, and DMEM + 10% FBS, For each nanomaterial, 1 mg was dispersed in 2 mL of each medium (500 μg/mL) by sonicating at 37 kHz for 1 h. The dispersions were photographed immediately after sonication, and then kept at 4°C for 3 months, at which point they were photographed again. A 1:10 dilution (50 μg/mL) was also prepared and the stability was evaluated over 25 days period.

#### Drug Release Studies

The *in vitro* DOX release from the CNT/HA-DMPE materials was evaluated at 37°C in PBS (pH 7.4) and acetate buffer (pH 5.5) by the dialysis method. Four milligram of each DOX/CNT/HA-DMPE material (HA MW 6.4, 17, 51, 200, and 1,500) was dispersed in 4 mL of PBS and transferred into dialysis bags (MWCO 3,500 Da) that were immersed in 35 mL of the release medium. At predetermined time intervals (0, 1, 3, 5, 24, 48, 72 h), 1 mL was taken and analyzed by UV-Vis spectroscopy to determine the amount of released DOX. The solution abstracted for UV-Vis analysis was immediately replaced with an equal volume of fresh medium.

### Biological Studies

#### Tumor Cell Lines and Cell Culture

The cell lines used were MDA-MB-231 (human breast adenocarcinoma) and A2780 (human ovarian carcinoma). MDA-MB-231 cells were grown in DMEM supplemented with 10% FBS, 0.03% L-glutamine, 2% penicillin and streptomycin. A2780 cells were maintained in RPMI 1640 containing 10% FBS, 0.03% of L-glutamine, 2% penicillin and streptomycin, and 50 μg/mL of gentamicin sulfate. Cells were maintained in a humidified incubator at 37°C in 5% CO_2_.

#### Cellular Uptake

A quantitative analysis of CNT/f-HA-DMPE cellular uptake was carried out on a C6 Flow Cytometer System (Accuri™ Cytometers, Milan, Italy). Briefly, MDA-MB-231 and A2780 cells were plated in 6-well culture plates (5 × 10^5^ cells/well) and incubated with CNT/f-HA-DMPE (10 μg/mL in PBS) for 3 and 24 h. Cells were washed twice with PBS, detached after each incubation period using a 0.05% trypsin−0.02% EDTA solution, and re-suspended in 500 μL PBS. They were then run on the flow cytometer, which considered 10,000 events, using 488 nm excitation to measure the intracellular CNT/f-HA-DMPE fluorescence (FL-1 channel, λ_ex_: 488 nm, λ_em_: 530 nm). Cell-associated fluorescence is expressed as integrated mean fluorescence intensity (iMFI); the product of the frequency of cells that are positive to CNT/f-HA-DMPE and the mean fluorescence intensity of the cells. Results are expressed as a ratio between the iMFI of treated and untreated cells.

#### Cell Proliferation Assay

The effect on cell growth inhibition was evaluated by the sulforhodamine B colorimetric proliferation assay (SRB) modified by Vichai and Kirtikara (Vichai and Kirtikara, 2006).

MDA-MB-231 and A2780 cells, maintained in culture as described above, were seeded at 3 × 10^4^ cells/well in 96 wells microtiter plates and incubated overnight to allow cellular adhesion. Various dilutions of DOX/CNT/HA-DMPE (expressed as drug concentration) and unloaded CNTs (expressed as CNTs concentration) were added in triplicate and incubated for 24, 48, and 72 h.

## Results and Discussion

### Synthetic Strategy

**Figure 1:**
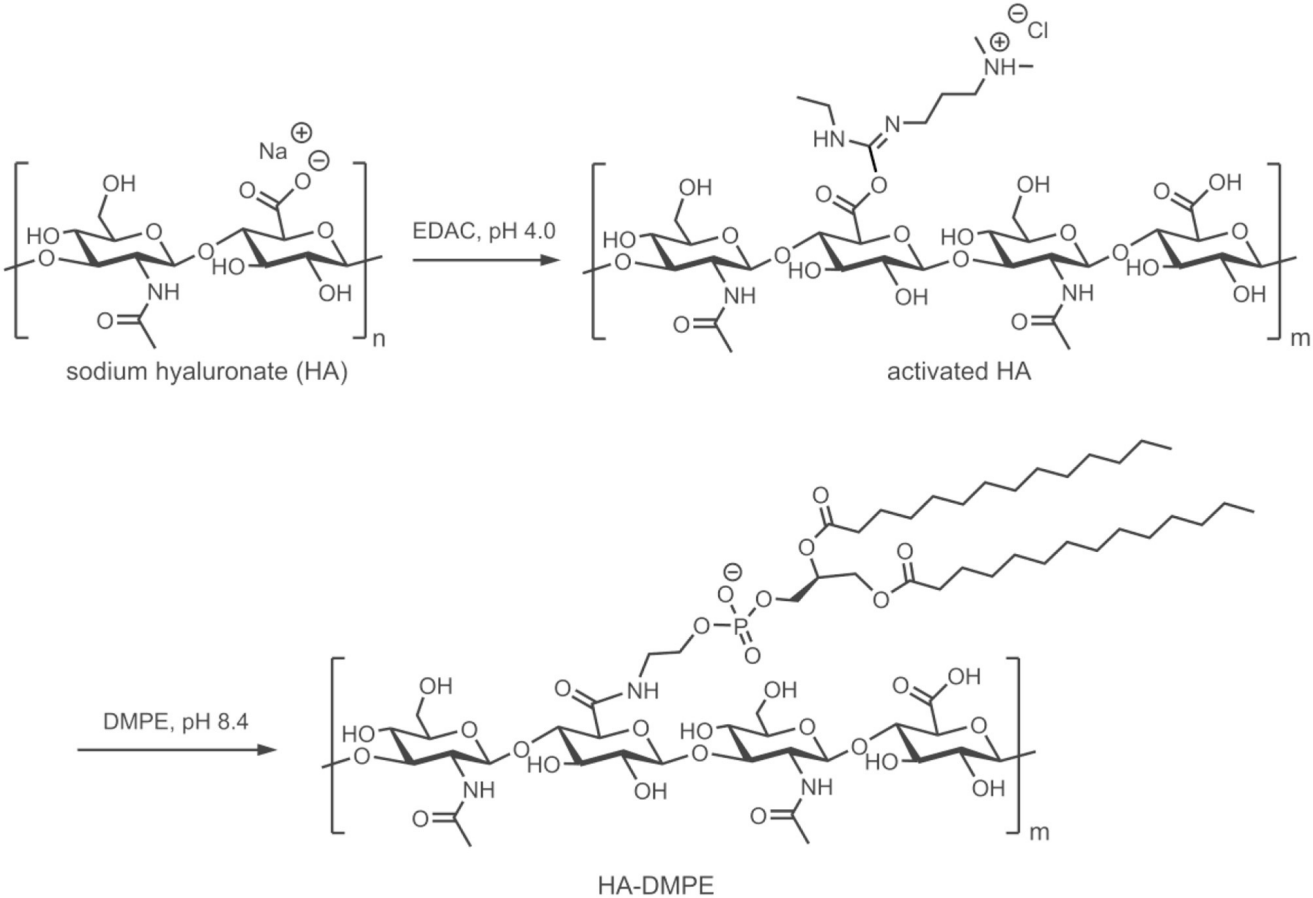
Schematic representation of the preparation of the HA-DMPE conjugate. The preparation of the HA-DPPE conjugate follows an identical procedure, with the exception that DPPE is used instead of DMPE.

HA of different MW (6.4, 17, 51, 200, and 1,500 kDa) was used to prepare a small library of conjugates with DMPE. After purification and characterization, the conjugates were used to functionalize CNTs with the aim of increasing their water dispersibility and biocompatibility, and to confer targeting ability toward CD44^+^ cells. The linkage between phospholipids and HA was obtained through amide formation mediated by the water-soluble carbodiimide derivative EDAC (Figure 1). In these conjugates, the phospholipid amino group is randomly linked to the carboxylic residues of HA, as previously reported (Surace et al., 2009; Cosco et al., 2017).

Conjugates were also prepared using DPPE instead of DMPE. Both conjugates permitted the functionalization of CNTs, confirming that the length of the phospholipid acyl chain does not influence the CNTs modification, as previously observed (Dvash et al., 2013). On the other hand, the different chemico-physical characteristics of the two phospholipids affected the reaction yield. Only the conjugates obtained with DMPE were used for further studies since they contained a higher amount of phospholipid linked to HA.

After purification by centrifugation and dialysis, the conjugates were lyophilized; ^1^H-NMR analysis (Supplementary Figure 1) confirmed the covalent linkage between DMPE and HA of different MW as previously reported (Cosco et al., 2017).

The amount of DMPE linked to HA was determined through a phosphorus assay. The degree of substitution (DS = mol DMPE/mol repeating unit × 100) ranged from 0.46 to 0.98%.

**Figure 2:**
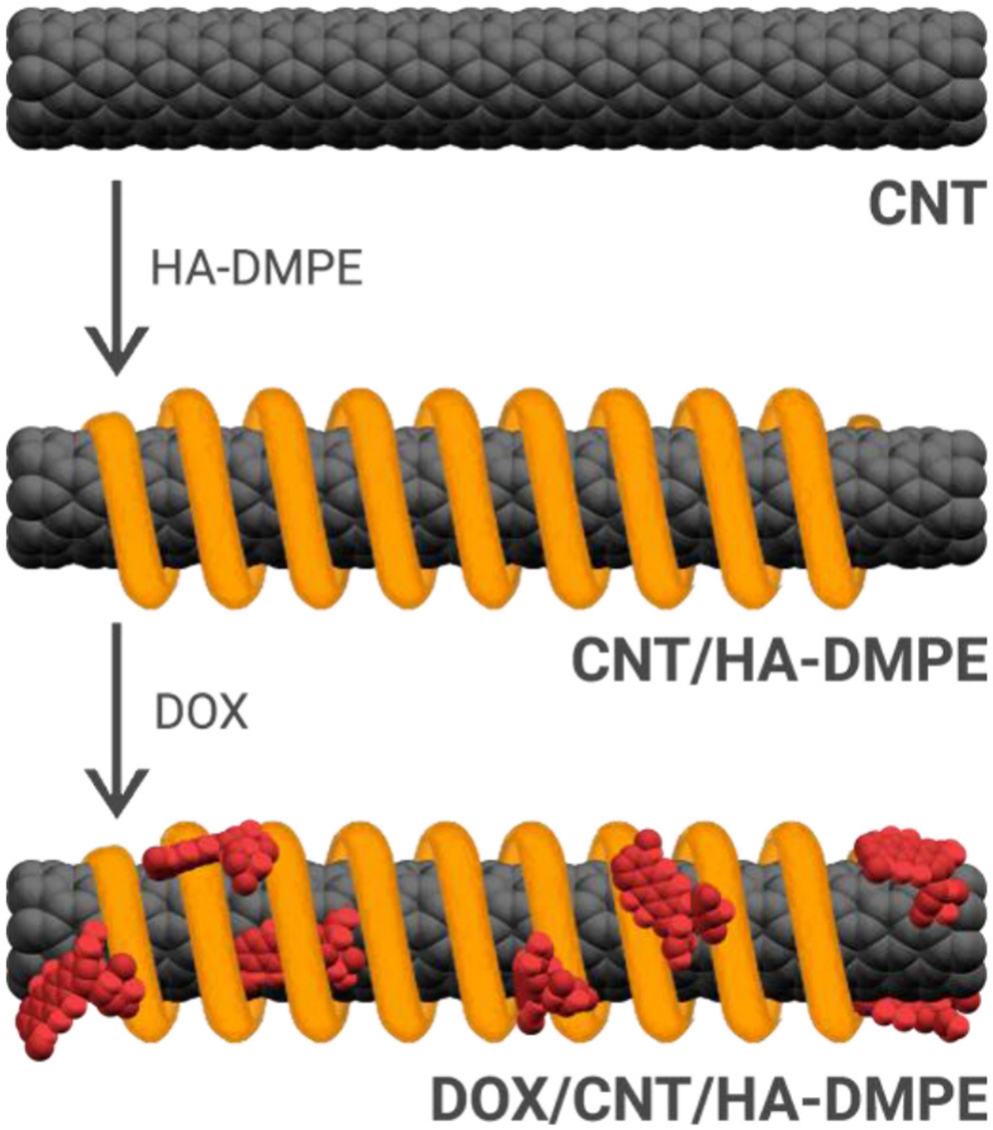
The non-covalent approach for the functionalization of CNTs with the HA-DMPE conjugate (orange helix) and DOX (red) to make a DOX/CNT/HA-DMPE system.

CNT/HA-DMPE systems were prepared through the non-covalent functionalization of CNTs with HA-DMPE conjugates of various HA MW. This improved the water dispersibility and biocompatibility of the CNTs. Then, the cationic anthracycline antibiotic DOX was loaded onto the CNT/HA-DMPE (Figure 2) via a π-π stacking interactions between the aromatic DOX and the sidewall of the CNTs, and van der Waals and hydrophobic interactions (Datir et al., 2012; Mehra et al., 2014; Wang and Xu, 2015).

Thus, CNTs were added to PBS suspension of the previously prepared HA-DMPE conjugates and sonicated for 3 h, then DOX was added, and the mixture was sonicated for a further 2 h. We preferred to add DOX in the second step of the reaction in order to reduce the risk of drug degradation related to a prolonged sonication time. The mixture was then purified by ultrafiltration in order to remove the unbound compounds. We have observed that a DOX: CNT/HA-DMPE ratio of 2:1 w/w allowed the complete drug loading in all the CNTs functionalized with the different HA-phospholipid conjugates previously prepared. On the other hand, when the amount of DOX was increased, unbounded drug was recovered during ultrafiltration. There was no linear correlation between the amount of unbounded DOX and the MW of the HA-DMPE conjugate; only in the case of non-functionalized CNTs, the amount of DOX loaded was doubled; a similar behavior was also observed by Yao et al. (2016).

A carbazole assay determined the quantity of HA non-covalently bound to the CNTs, for all HA-functionalized CNT materials. The results indicated that it was identical to the quantity added in the functionalization reaction, meaning that all of the conjugate was adsorbed onto the CNT surface in each case.

### Chemico-Physical Characterization

#### Transmission Electron Microscopy

**Figure 3:**
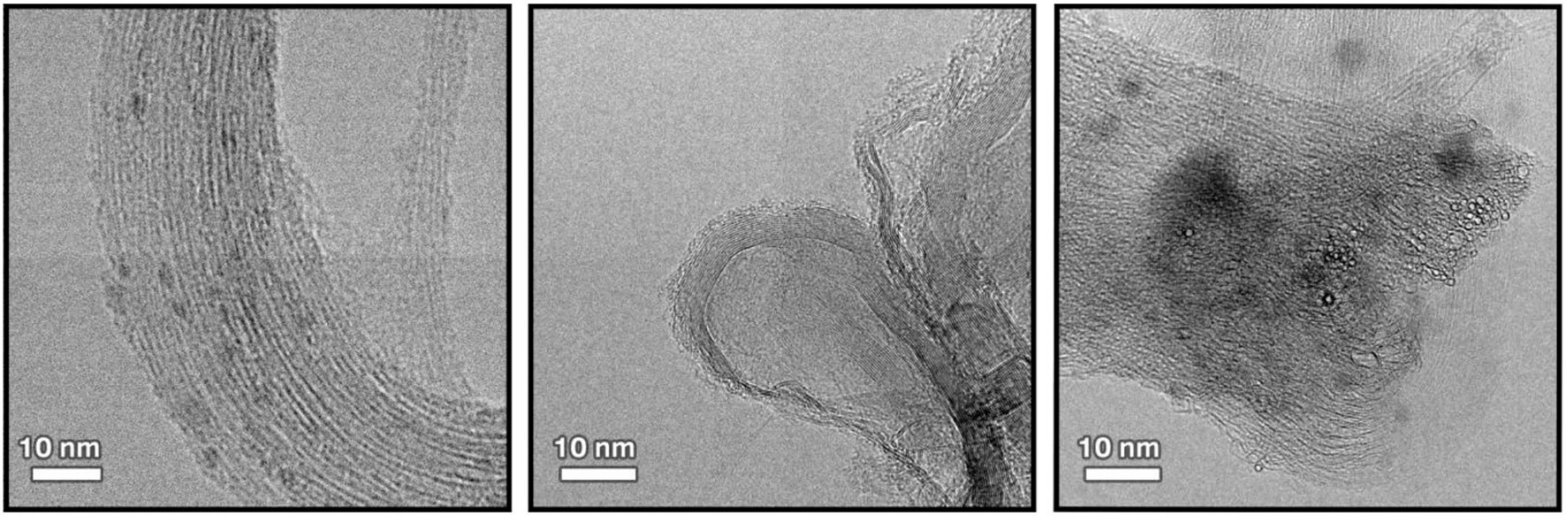
Representative TEM images at x300K magnification of pristine CNTs (left); CNT/HA_200_-DMPE (middle); and CNT/HA_6.4_-DMPE (right).

The structure and morphology of the functionalized CNTs was analyzed by TEM. Representative TEM images of pristine CNTs and CNT/HA-DMPE are depicted in Figure 3. The graphitic structure of the nanotube is evident, indicating that the process of coating CNTs with the HA-DMPE conjugate does not affect the structural integrity of the nanomaterial. The CNTs are clearly visualized. The polymeric material on the CNT surface in the nanocarriers HA-DMPE cannot be readily seen as it is in an amorphous form. Compared to the pristine CNTs, the CNT/HA_6.4_-DMPE and CNT/HA_200_-DMPE did not exhibit appreciable differences in morphology or aggregation.

#### Thermogravimetric Analysis

**Figure 4:**
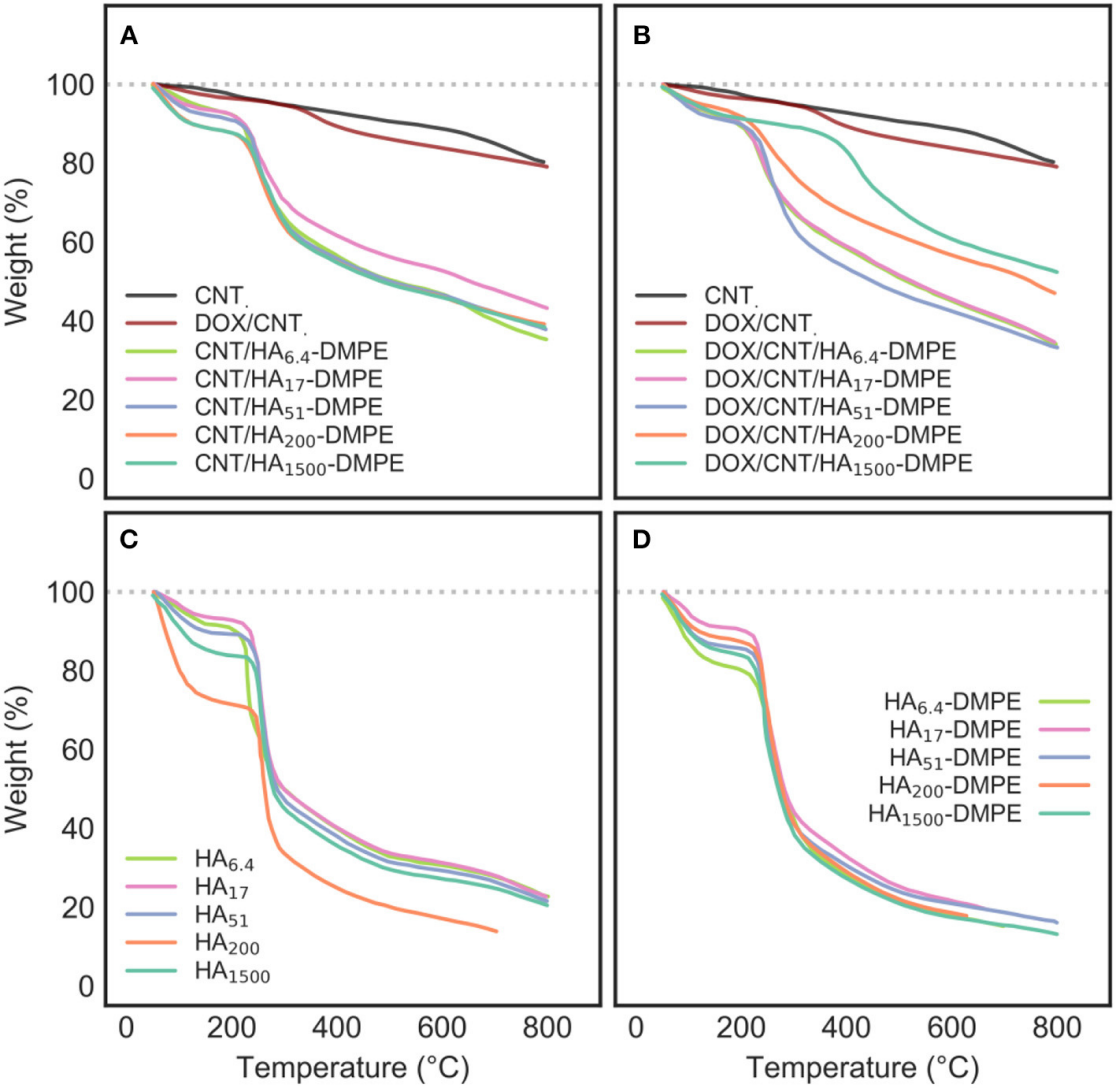
TGA profiles of **(A)** CNT/HA-DMPE; **(B)** DOX/CNT/HA-DMPE; **(C)** HA; and **(D)** HA-DMPE analyzed at different chain lengths of HA, including 6.4, 17, 51, 200, and 1,500 kDa. The TGA profiles of CNT and DOX/CNT have been included in both **(A,B)** for reference.

TGA was used to characterize CNT, DOX/CNT, CNT/HA-DMPE, and DOX/CNT/HA-DMPE, as well as HA-DMPE and HA for comparative purposes (Figure 4).

Pristine CNTs show a typical, low weight loss, which is associated with the defects and oxidized functional groups present on the surface.

In contrast, CNT/HA-DMPE and DOX/CNT/HA-DMPE lost a considerable amount of their weight, demonstrating the success of surface functionalization with HA-DMPE and DOX. Furthermore, the main stage of weight loss in CNT/HA-DMPE and DOX/CNT/HA-DMPE occurs at an almost identical temperature (270°C) as observed for both HA and HA-DMPE, with a small shift toward higher temperature for the conjugated CNTs; a further indication of the correct derivatization.

Notably, DOX/CNT has a slightly-higher weight loss profile than that of pristine CNTs, which can be ascribed to the degradation of DOX non-covalently linked to CNTs surface. In addition, thermogravimetric DOX/CNT/HA-DMPE profile shows a significant more pronounced weight loss compared with DOX/CNT ones. The presence of HA-DMPE on CNTs surface may enhance the loading ability of the nanocarrier, probably because of the additional interaction between polymeric chains and DOX.

However, the interaction between CNT/HA-DMPE and DOX to give DOX/CNT/HA-DMPE seems to occur by displacing some HA-DMPE chains from the nanotube surface. The weight loss of DOX/CNT/HA-DMPE is equal, or lower (depending from HA size), than CNT/HA-DMPE, indicating that the interaction between aromatic rings of DOX, the graphitic surface the CNT, and the linked DMPE residue requires the displacement of some hindering HA-DMPE chains on the particle outer layer.

#### Zeta Potential

Zeta potential (ZP) analysis (Table 1) confirmed the presence of HA on the CNTs surface. The measured ZP has an inversely proportional relationship with HA MW, decreasing as the MW increases. This is due to the multiple carboxyl groups present on HA. Moreover, when the positively charged DOX was added, the zeta potential increased, indicating the successful adsorption of the drug onto the nanotubes.

**Table 1:** Zeta potential (ZP) values of the different functionalized CNTs.

**Sample**	**ZP ± S.D. (mV)**
CNTs	−18.8 ± 0.7
DOX/CNTs	−8.6 ± 0.4
CNT/HA_6.4_-DMPE	−21.4 ± 1.1
DOX/CNT/HA_6.4_-DMPE	−2.91 ± 1.0
CNT/HA_17_-DMPE	−25.9 ± 1.2
DOX/CNT/HA_17_-DMPE	−7.3 ± 1.4
CNT/HA_51_-DMPE	−28.7 ± 1.9
DOX/CNT/HA_51_-DMPE	−9.6 ± 1.0
CNT/HA_200_-DMPE	−34.1 ± 0.7
DOX/CNT/HA_200_-DMPE	−19.8 ± 1.0
CNT/HA_1500_-DMPE	−48.3 ± 0.5
DOX/CNT/HA_1500_-DMPE	−41.0 ± 0.9

#### UV-Vis Absorption and Fluorescence Spectroscopy

**Figure 5:**
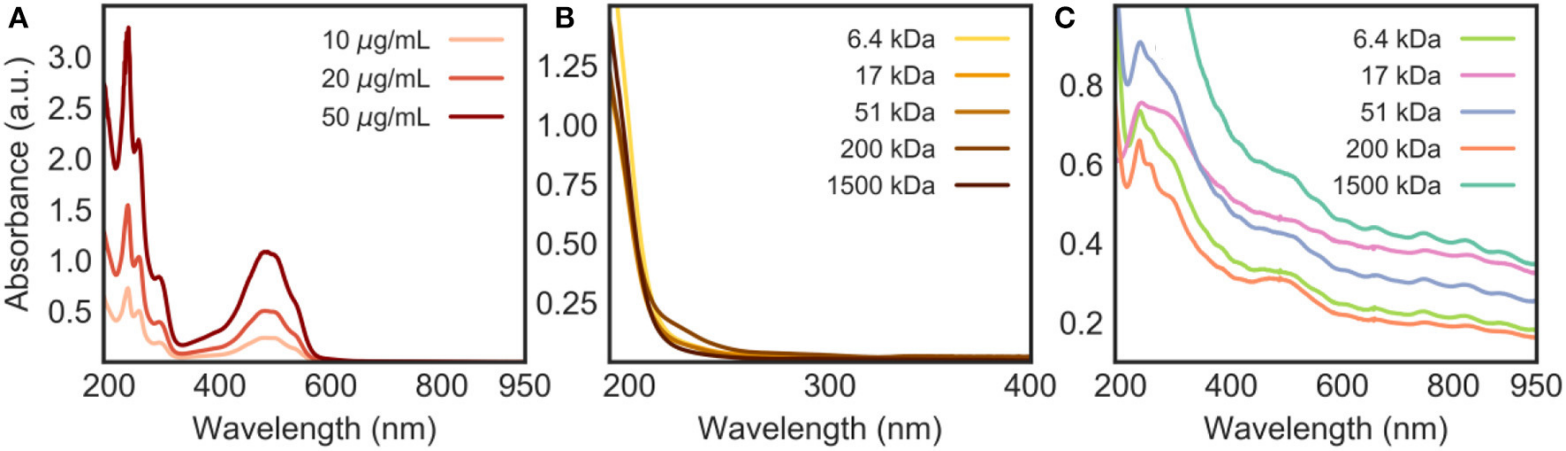
UV-Vis absorption spectra in water of **(A)** DOX at various concentrations (10, 20 and 50 μg/mL); **(B)** HA-DMPE (50 μg/mL); and **(C)** DOX/CNT/HA-DMPE (10 μg/mL). Materials involving HA were analyzed at various chain lengths (6.4, 17, 51, 200, and 1,500 kDa).

The loading of DOX onto the CNT/HA-DMPE nanohybrids was confirmed by UV-Vis and fluorescence spectroscopy analysis. DOX has a characteristic absorption peak at 480 nm (Figure 5A); given that HA-DMPE does not absorb in the visible region (Figure 5B), the presence of a peak at 480 nm in DOX/CNT/HA-DMPE (Figure 5C) confirms the presence of the drug in the nanomaterial. Also, it is interesting to note that the Van Hove singularities (Kim et al., 1999) of the CNT can be seen in the absorption spectrum of DOX/CNT/HA-DMPE (Figure 5C). These are points where the density of states (i.e., the number of quantum states that electrons inside the system can take) is not differentiable (Kataura et al., 1999; Ryabenko et al., 2004). A number of these Van Hove singularities can be seen as small features above 600 nm (Figure 5C). Their presence further indicates that the intrinsic structure of the CNTs is not affected by the loading of HA-DMPE and DOX.

From the absorption spectrum of DOX/CNT/HA-DMPE (Figure 5C), it can also be concluded that 1,500 kDa HA is the best of the five MWs analyzed for dispersing the nanomaterial, as evidenced by the highest absorption of the nanomaterial across the spectrum.

**Figure 6:**
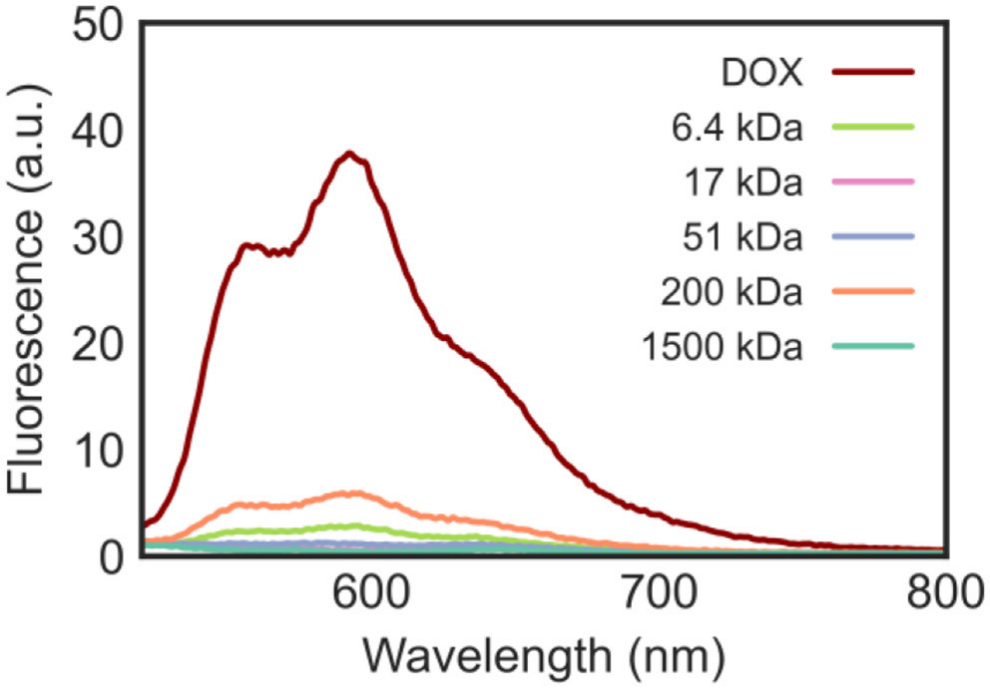
Emission spectra in water at λ_ex_ 480 nm of DOX (10 μg/mL) and DOX/CNT/HA-DMPE (20 μg/mL) at various HA chain lengths (6.4, 17, 51, 200, and 1,500 kDa).

When excited by light in the blue-visible range, DOX fluoresces due to its highly conjugated structure. The emission profile of DOX (Figure 6) shows that, when excited at λ_ex_ 480 nm, the anticancer drug emits at λ_max_ 592 nm with the strongest intensity. When the fluorescent DOX was adsorbed on the surface of the nanomaterials, the fluorescence signal decreased due to the proximal distance and energy transfer. The emission spectra of the DOX/CNT/HA-DMPE nanocomposites are shown in Figure 6 and confirm the successful loading of the drug onto CNT/HA-DMPE.

#### Stability Studies

**Figure 7:**
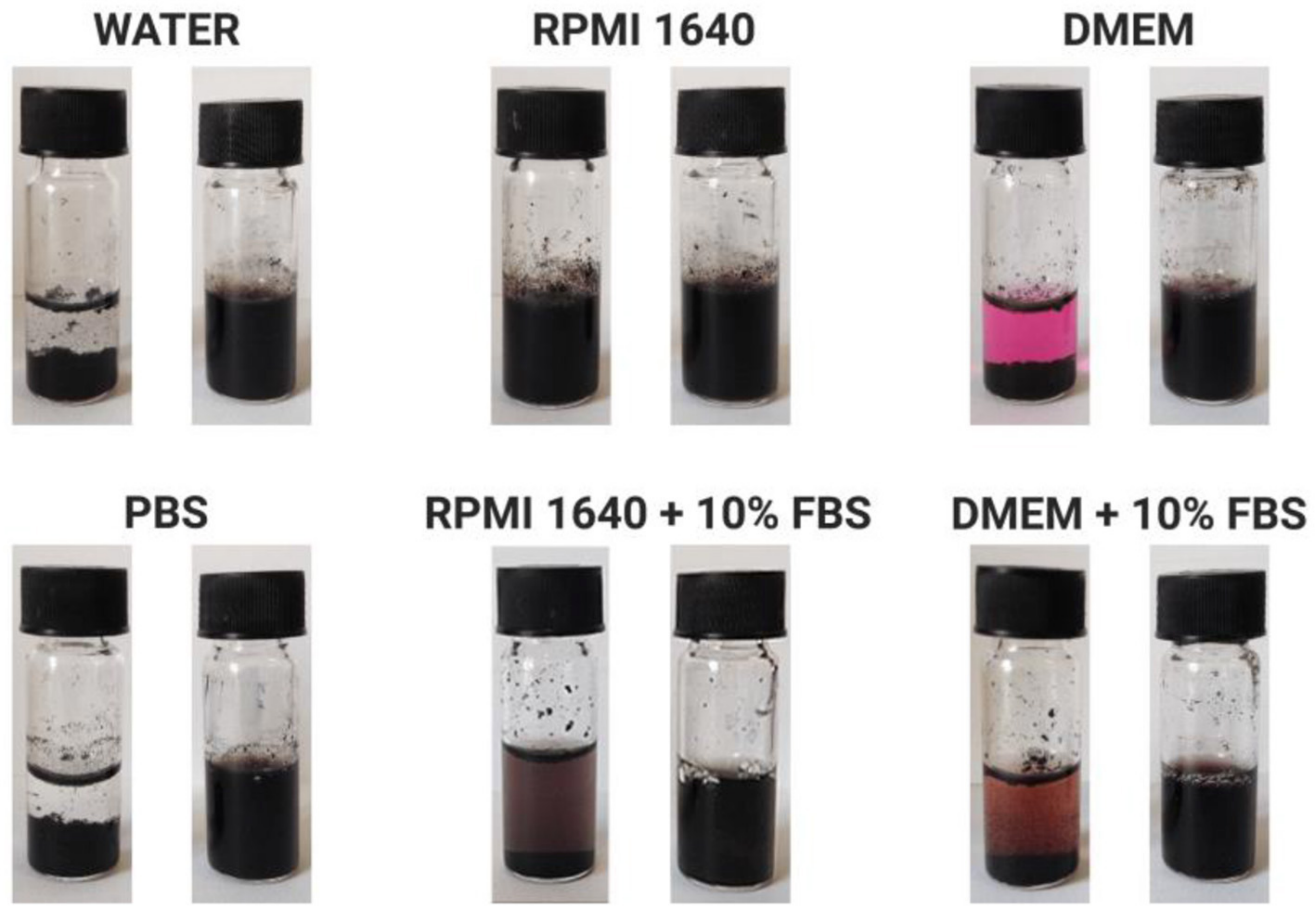
Representative images of DOX/CNT (left) and DOX/CNT/HA-DMPE (right) dispersion stability after 3 months at 4°C in various biological fluids at a concentration of 500 μg/mL. Representative images are of DOX/CNT/HA_200_-DMPE. Other HA MW showed similar results [data not shown].

The modification of DOX/CNTs with HA-DMPE conjugates of different MW significantly increased their water dispersity and stability (Figure 7 and Supplementary Figure 2). The DOX/CNT/HA-DMPE of different HA MW were well-dispersed and stable without aggregation in different media for over 3 months. In contrast, DOX/CNT was not dispersible in any of the tested media. The media chosen for this experiment were those used for the cell lines culture. These results confirm that the HA-DMPE conjugate imposes excellent dispersibility and stability onto the CNT systems.

#### Drug Release Studies

**Figure 8:**
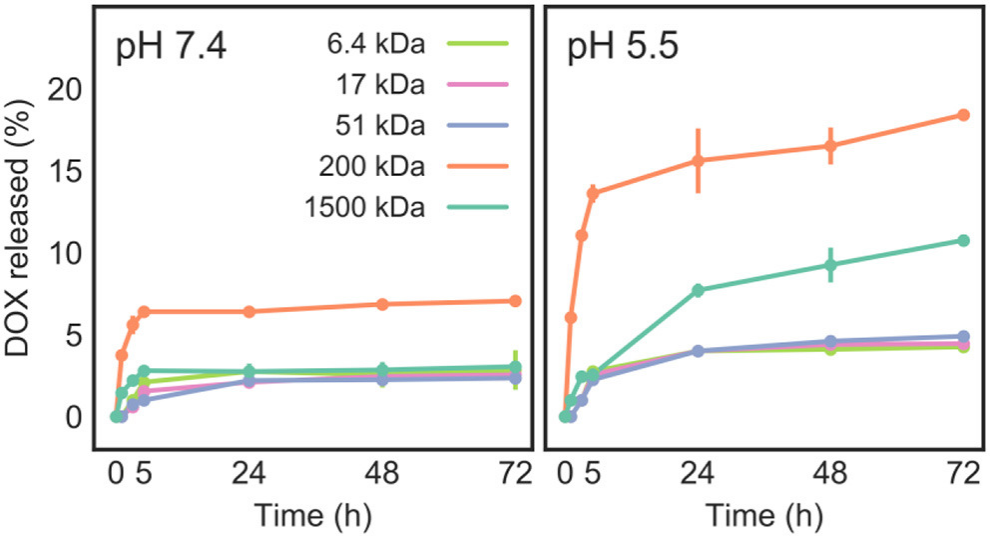
Release of DOX from DOX/CNT/HA-DMPE at pH 7.4 and 5.5, as measured at 37°C over a 72 h period.

The *in vitro* release profile of DOX from DOX/CNT/HA-DMPE was investigated at 37°C at two different pH values (7.4 and 5.5), which represent the physiological condition and acidic microenvironment of both tumor tissue and lysosomes, respectively (Figure 8). We observed that the DOX release rate is faster at pH 5.5 than at pH 7.4. After 72 h, at most, around 20% of DOX was released at acidic pH, and only 5% of DOX was released at physiological pH. The overall low DOX release at both pH values is probably due to steric hindrance and chain entanglement of the HA coating. Moreover, once internalized into tumor cellsCNTs will further release DOX in the acidic compartment of lysosomes, given the enhanced hydrophilicity and solubility of DOX in low pH environments (Wang and Xu, 2015).

As the pH was lowered, drug release was increased, suggesting an endogenous pH-responsive mode of controlled drug release. This means that DOX will be selectively released in acidic microenvironments, such as that in tumors, while at physiological pH, the drug will remain preferentially loaded on the CNT surface. The increase of DOX release at low pH is due to the partial protonation of DOX amino groups, which increases its hydrophilicity and does not favor the π-π stacking interactions between the drug and CNTs, facilitating a partial DOX detachment from the carrier (Wong et al., 2013; Wang and Xu, 2015).

The results (Figure 8) also presented a drug release dependency on the MW of the HA-DMPE conjugate. The highest drug release (about 7% at pH = 7.4 and 18% at pH = 5.5) was observed for DOX/CNT/HA_200_-DMPE, indicating that it is the ideal HA MW of the five investigated variants. In the case of higher MW DOX/CNT/HA_1500_-DMPE, a reduction of DOX release was observed. Given that the amount of loaded drug is similar for all the samples, this behavior could be attributed to the high MW of the polymer that hampers the drug release by steric hindrance.

### Biological Studies

#### Cellular Uptake

**Figure 9:**
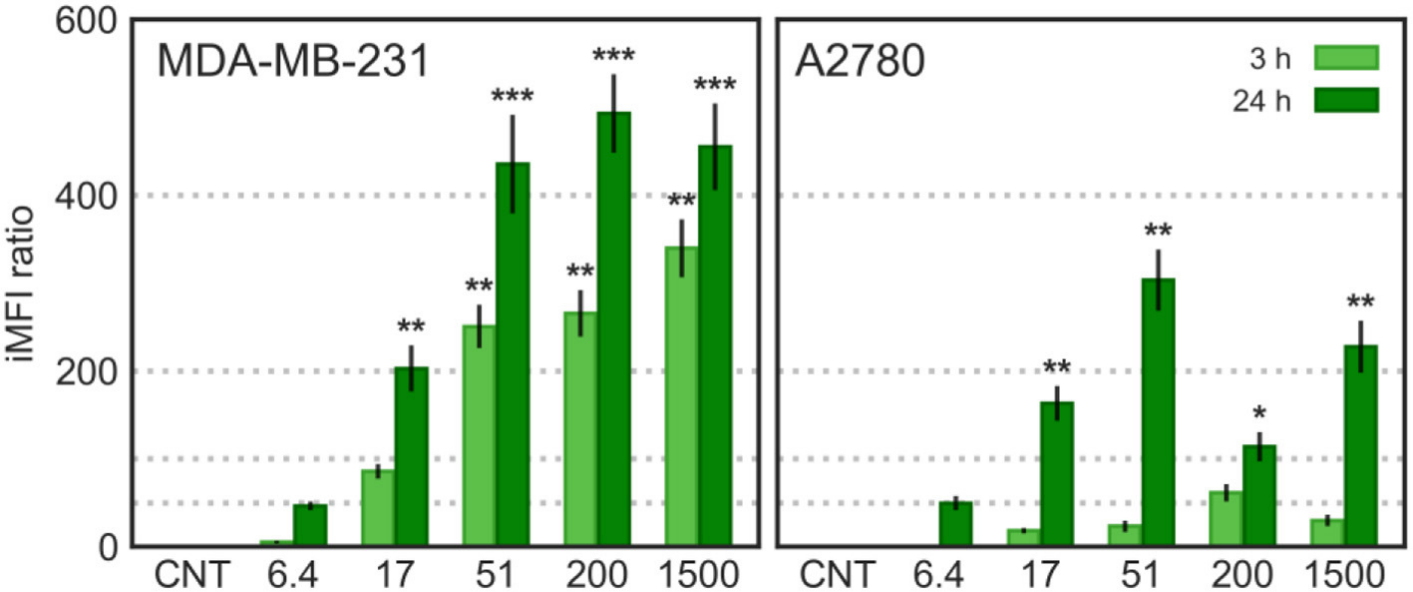
Cytofluorimetric cellular uptake; MDA-MB-231 and A2780 cells were incubated with CNTs and CNT/f-HA-DMPE of increasing HA MW (6.4, 17, 51, 200, and 1,500 kDa) at the same concentration (10 μg/mL) for 3 and 24 h and fluorescence was evaluated on FL-1 channel (λ_ex_: 488 nm, λ_em_: 530 nm). Statistical significance vs. CNTs: *p < 0.05 **p < 0.01, ***p < 0.001.

The HA receptor CD44, a ubiquitous transmembrane cell surface protein, is expressed at low levels on the surface of several normal cells and overexpressed in many cancer cells (Sneath and Mangham, 1998). We have recently reported that the MDA-MB-231 (human breast adenocarcinoma) cells display high expression of CD44 and that the A2780 (human ovarian carcinoma) cells did not express a detectable amount of CD44 (Ricci et al., 2018). Thus, to evaluate the cellular uptake and the cytotoxic activity of the previously prepared CNT/HA-DMPE, MDA-MB-231 and A2780 cells were chosen as CD44^+^ and CD44^−^ cells, respectively. For these studies, fluorophore-labeled conjugates (f-HA-DMPE) were used; the cellular uptake of CNTs and CNT/f-HA-DMPE at increasing HA MW was quantitatively evaluated by flow cytometry in the CD44^+^ and CD44^−^ cell lines (Figure 9).

In MDA-MB-231 (CD44^+^) cells, the CNTs uptake was significantly increased over time and increasing HA MW. In A2780 (CD44^−^) cells, a significant increase in CNT/f-HA-DMPE cellular uptake was observed only after 24 h incubation, to an extent independent from HA MW. Moreover, comparing the iMFI ratio obtained in the two cell lines, the highest value was observed with CNT/f-HA_200_-DMPE in MDA-MB-231 cells and with CNT/f-HA_51_-DMPE in A2780 cells after 24 h incubation. The uptake level observed in the CD44^−^ cell line after 24 h was significantly lower than that observed in the CD44^+^ cell line after the same incubation time. Likewise, the levels reached in the CD44^+^ cell line after just 3 h incubation were similar to that in the CD44^−^ cell line after 24 h incubation.

These results are consistent with the idea that MDA-MB-231 cells uptake CNT/f-HA-DMPE through the CD44 mediated endocytosis pathway, highlighting their potential use as an efficient approach for tumor-targeting treatments. Moreover, in A2780 cells, aspecific mechanisms over receptor mediated-pathways can have a role in the CNT/f-HA-DMPE uptake. Similar results were also obtained by us evaluating the cellular uptake of HA decorated carbon nano-onions by confocal live-cell imaging (d'Amora et al., 2020).

#### Effects on Cell Proliferation

**Figure 10:**
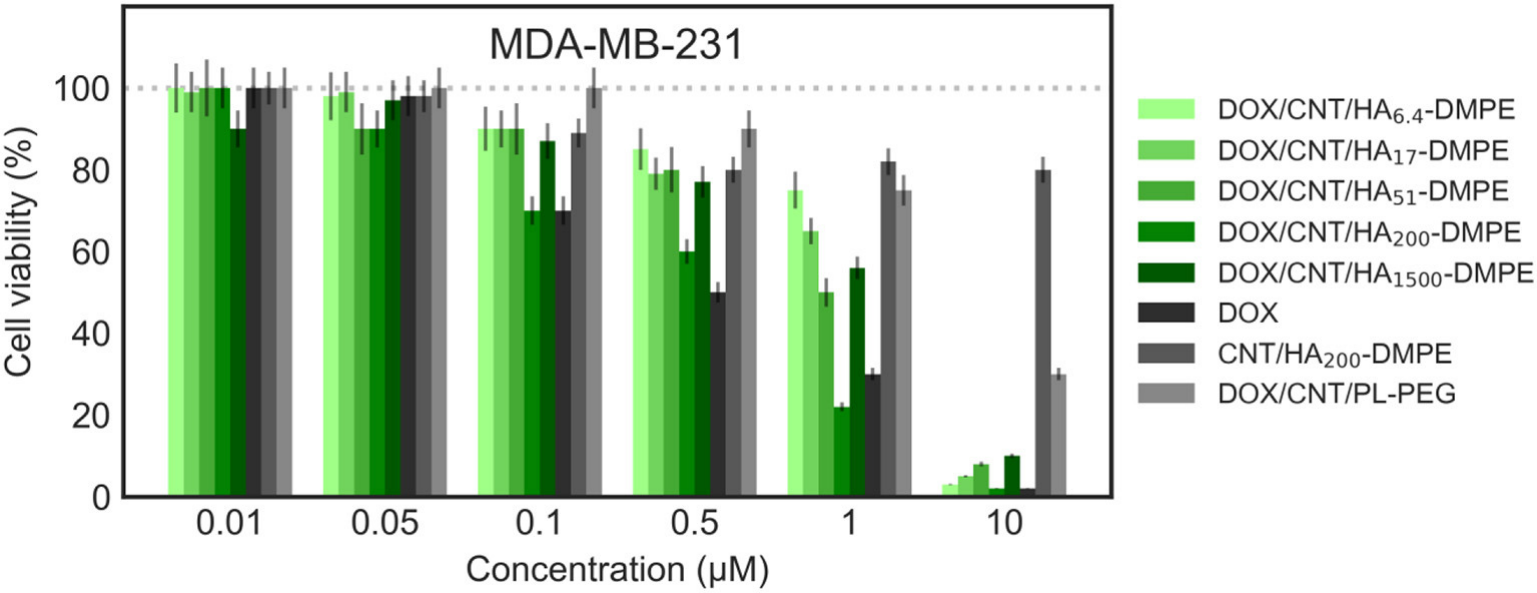
Viability of the MDA-MB-231 (CD44^+^) cell line when treated with various formulations at different concentrations for 24 h. Cell viability was determined using the sulforhodamine B (SRB) assay as previously described in material and methods section.

**Figure 11:**
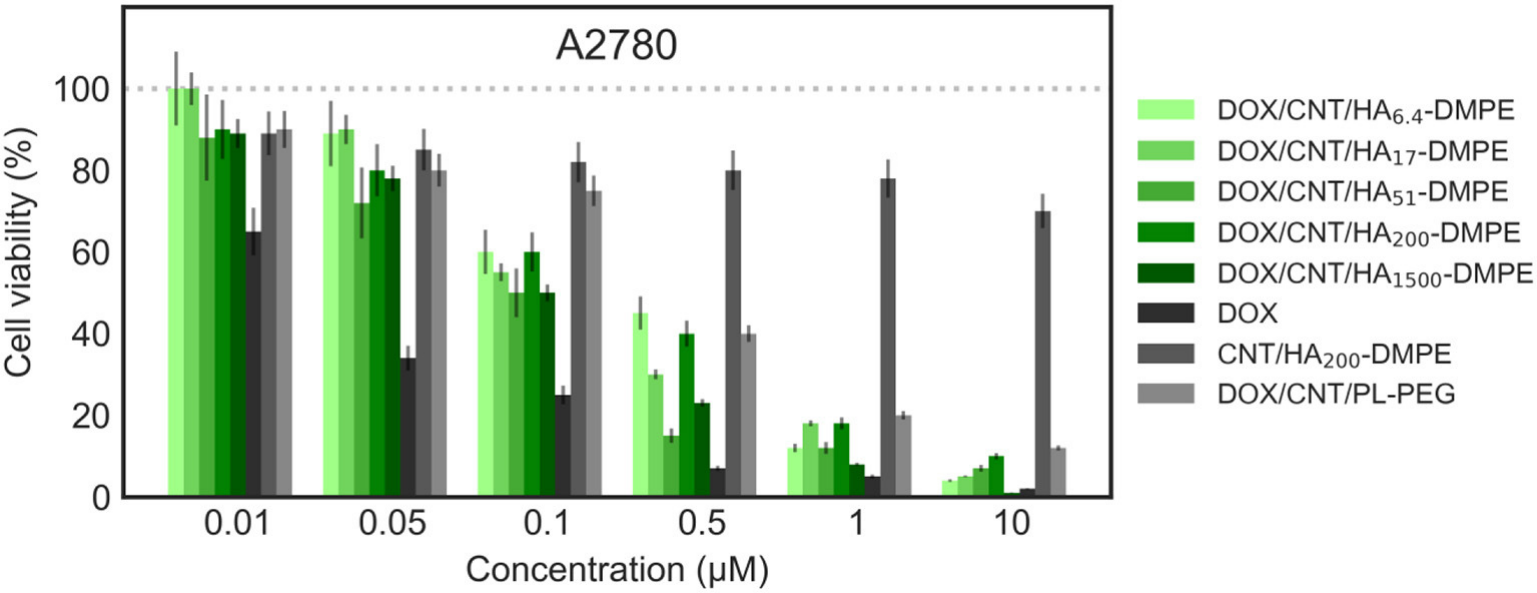
Viability of the A2780 (CD44^−^) cell line when treated with various formulations at different concentrations for 24 h. Cell viability was determined using the sulforhodamine B (SRB) assay as previously described in material and methods section.

The *in vitro* cytotoxicity of the different preparations was evaluated on MDA MB231 (CD44^+^) and A2780 (CD44^−^) cells at three different incubation times; 24 (Figures 10, 11), 48 and 72 h.

On both cell lines, CNT/HA-DMPE of different HA MW did not present significant effect on cell proliferation, while DOX showed a marked difference in activity (IC_50_ 0.5 μM on MDA-MB- 231 and 0.032 μM on A2780 cell lines). A linear increase of cytotoxicity was observed by increasing incubation time (higher for A2780 with 1 log decrease from 24 to 48 h). Thus, the comparison of the cytotoxic activity reported in Figures 10, 11 represents a 24 h incubation time. On target cells (MDA-MB-231), the DOX/CNT/HA_200_-DMPE displayed higher cytotoxicity, comparable to DOX, while both lower and higher HA MW seemed to release the drug less efficiently. Increasing incubation time reduced this difference (data not shown).

Due to their scarce water dispersibility, pristine CNTs cannot be tested on cell lines. For this reason, the 1,2-distearoyl-*sn*-glycero-3-phosphoethanolamine-N-[amino(polyethylene glycol)-2000] conjugate (PL-PEG) was employed to suspend DOX/CNTs (DOX/CNT/PL-PEG) following a procedure described elsewhere (Liu et al., 2009). On A2780 cells, no significant difference between PEG or HA decorated CNTs has been observed. On the contrary, on CD44^+^ cells, a significative difference was observed, which appeared more evident in comparison to DOX/CNT/HA_200_-DMPE, confirming the cytofluorimetric results about the involvement of the HA receptor in the cellular uptake, and consequent, cytotoxicity in CD44^+^ cells.

## Conclusion

In this study, a small library of HA-DMPE conjugates was prepared using HA of different MW (6.4, 17, 51, 200, and 1,500 kDa). These were non-covalently functionalized onto CNTs, which were further loaded with DOX. TGA, ZP, UV-Vis absorption and fluorescence spectroscopy confirmed the DOX loading and the HA-DMPE coating of CNTs. Moreover, due to the HA coating, the nanostructures were easily dispersed and stable in water and biological media. The data obtained by the cellular uptake and cell proliferation experiments indicated that CNT/HA_200_-DMPE manifested the best active targeting ability among all the conjugates. Drug release studies also suggested an endogenous pH-responsive mode for site-specific drug release which is of utmost importance for therapeutic applications in tumors.

Altogether, the results suggest the possible use of our conjugate, HA-DMPE, for an easy approach to improve the use of CNTs for diagnostic and therapeutic purposes.

Of all the CNT-based drug delivery systems prepared in this study, DOX/CNT/HA_200_-DMPE proved to be the most viable nanocarrier. The underlying material, CNT/HA_200_-DMPE, showed good biocompatibility, was easy to prepare, and was highly dispersible in water and biological media. Furthermore, biological studies confirmed that the system possessed good targetability toward CD44 overexpressing cells. The nanocarrier, DOX/CNT/HA_200_-DMPE, also possesses a “smart release” feature, as identified through drug release studies. The release of DOX has been found to be endogenously stimulated at acidic pH, meaning that the DOX/CNT/HA_200_-DMPE nanocarrier can selectively deliver the drug to tumor cells, whilst avoiding premature drug release in tissues at physiological pH.

## Data Availability Statement

The original contributions generated for the study are included in the article/Supplementary Material, further inquiries can be directed to the corresponding author.

## Author Contributions

SA conceived the idea, supervised the chemical synthesis of compounds and the biological experiments, and wrote the manuscript. MB performed spectroscopic measurements and wrote the manuscript. AB performed TGA measurements and analysis. DZ and LS performed the biological experiments. LS, PM, and FD analyzed the biological data. FD wrote the introduction. BS contributed to the discussion of the results and to the revision of the manuscript. SG conceived the idea, performed TEM, supervised the chemico-physical characterization of the CNTs, and contributed to the writing of the manuscript. All authors contributed to the article and approved the submitted version.

## Conflict of Interest

The authors declare that the research was conducted in the absence of any commercial or financial relationships that could be construed as a potential conflict of interest. The reviewer PP declared a past co-authorship with one of the authors SG to the handling Editor.
