# Supplementary material for: Effects of the Molecular Weight of Hyaluronic Acid in a Carbon Nanotube Drug Delivery Conjugate
Source: Front Chem. 2020 Dec 14;8:578008. doi: 10.3389/fchem.2020.578008 (PMC7767879; doi:10.3389/fchem.2020.578008)
Supplement: Supplementary file 1 [file Data_Sheet_1.pdf]

Supplementary Material

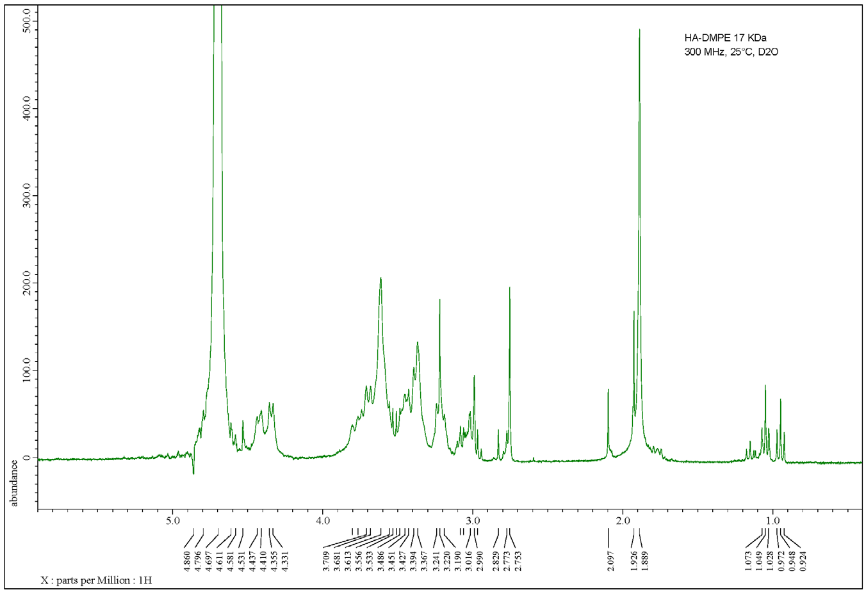

Supplementary Figure 1. Representative ^{1}H-NMR spectra of HA_{17}-DMPE measured in D_2O.

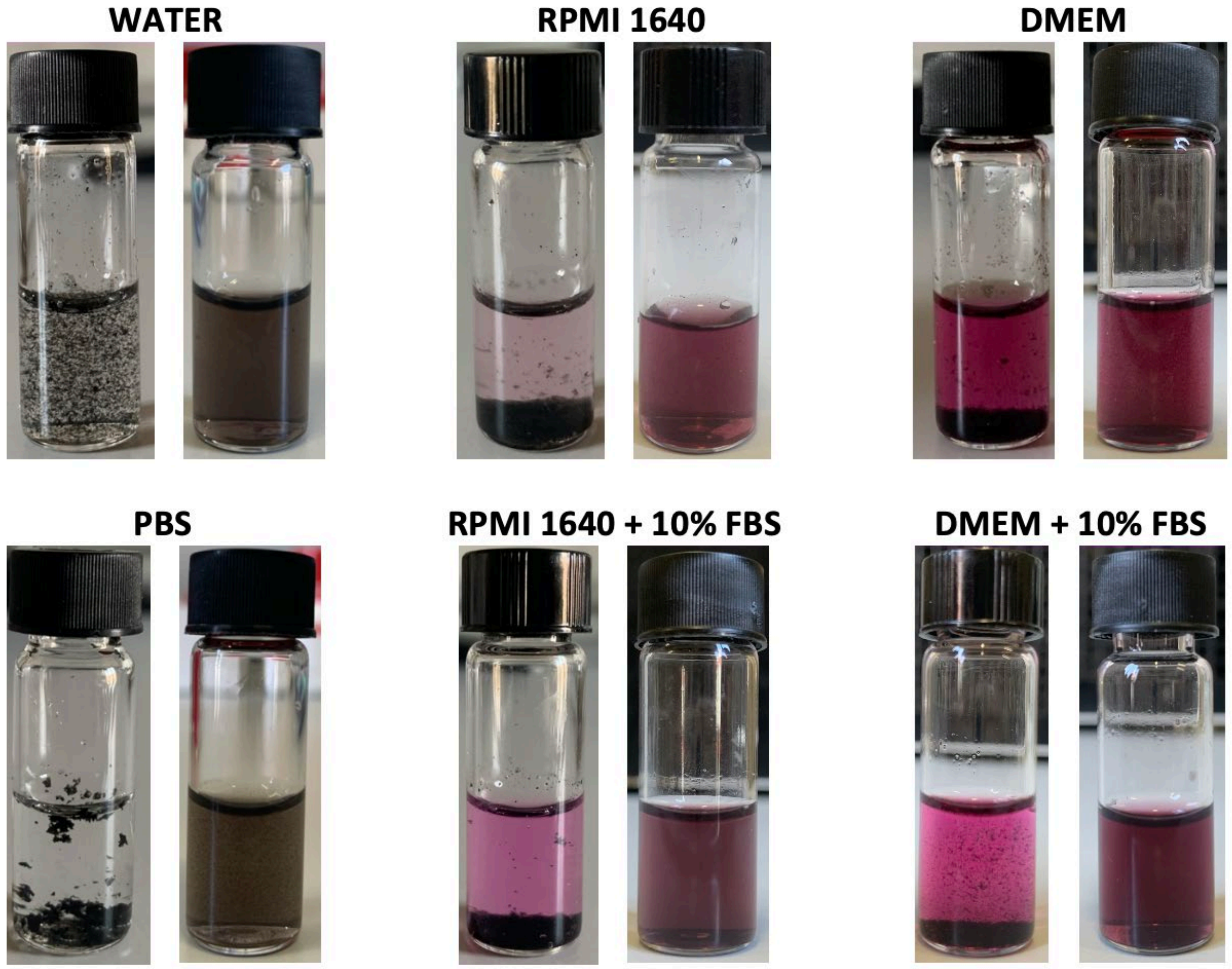

Supplementary Figure 2. Representative images of DOX/CNT (left) and DOX/CNT/HA_{200}-DMPE (right) dispersion stability after 25 days at 4°C in various biological fluids at a concentration of 50 μg/mL.
